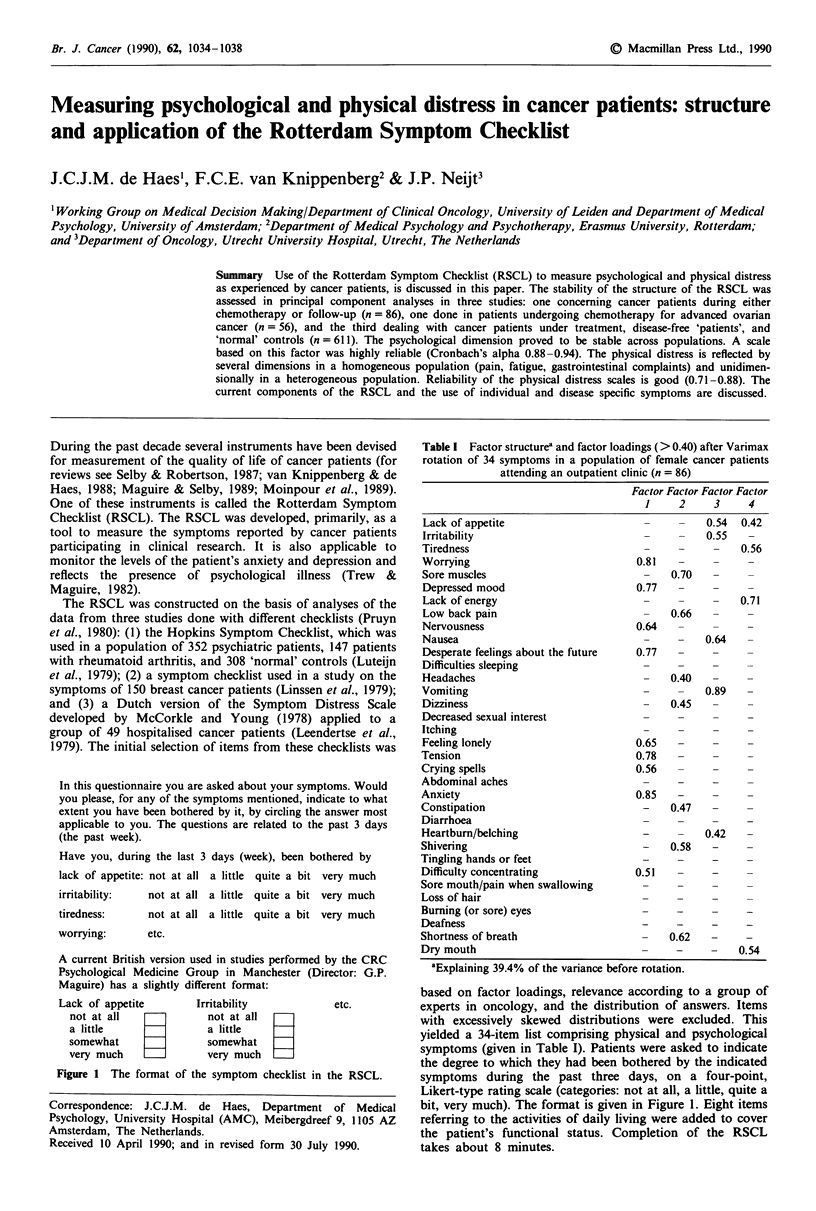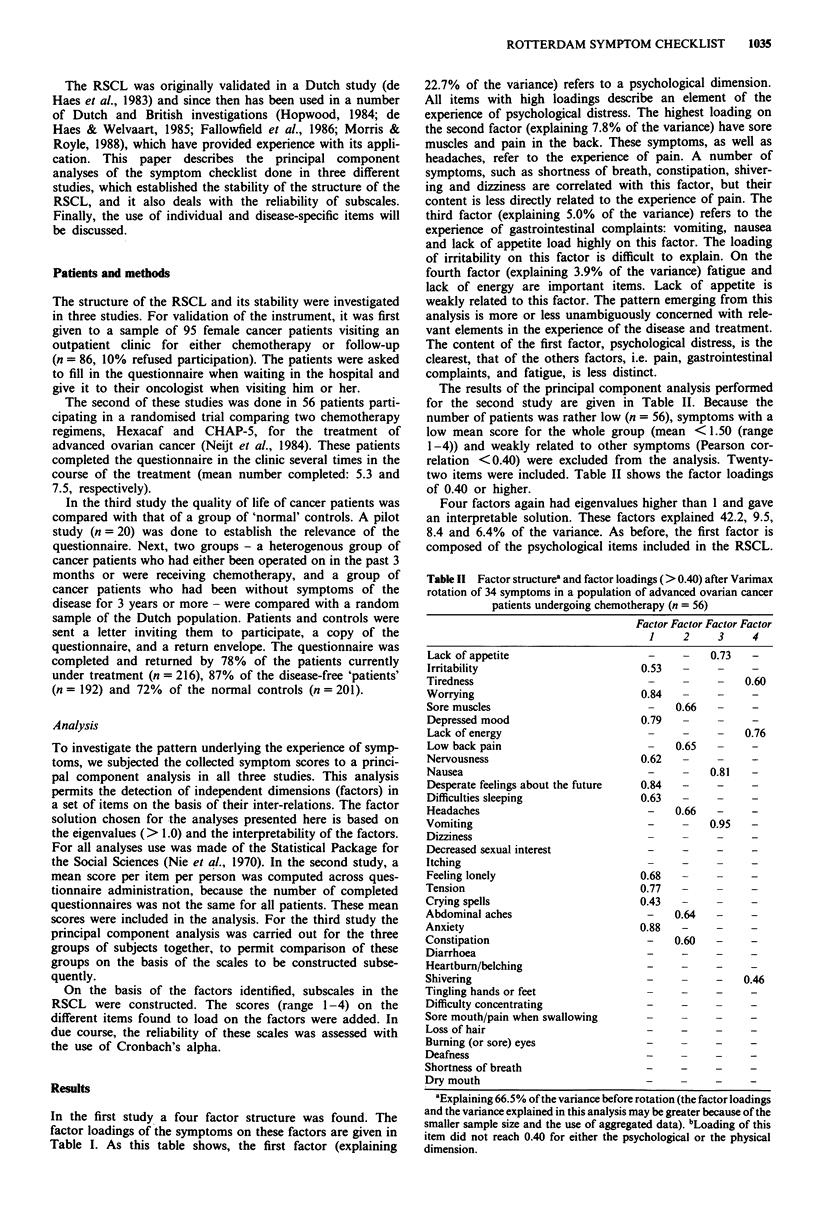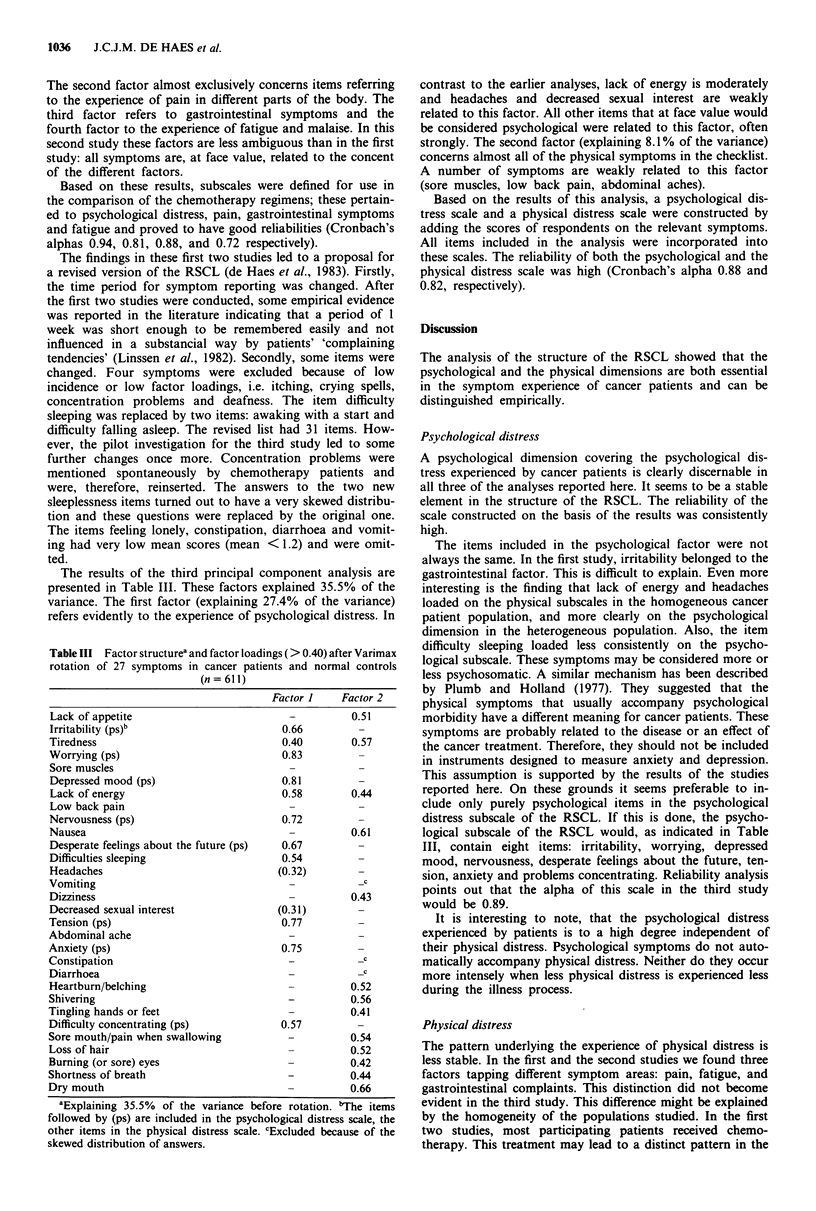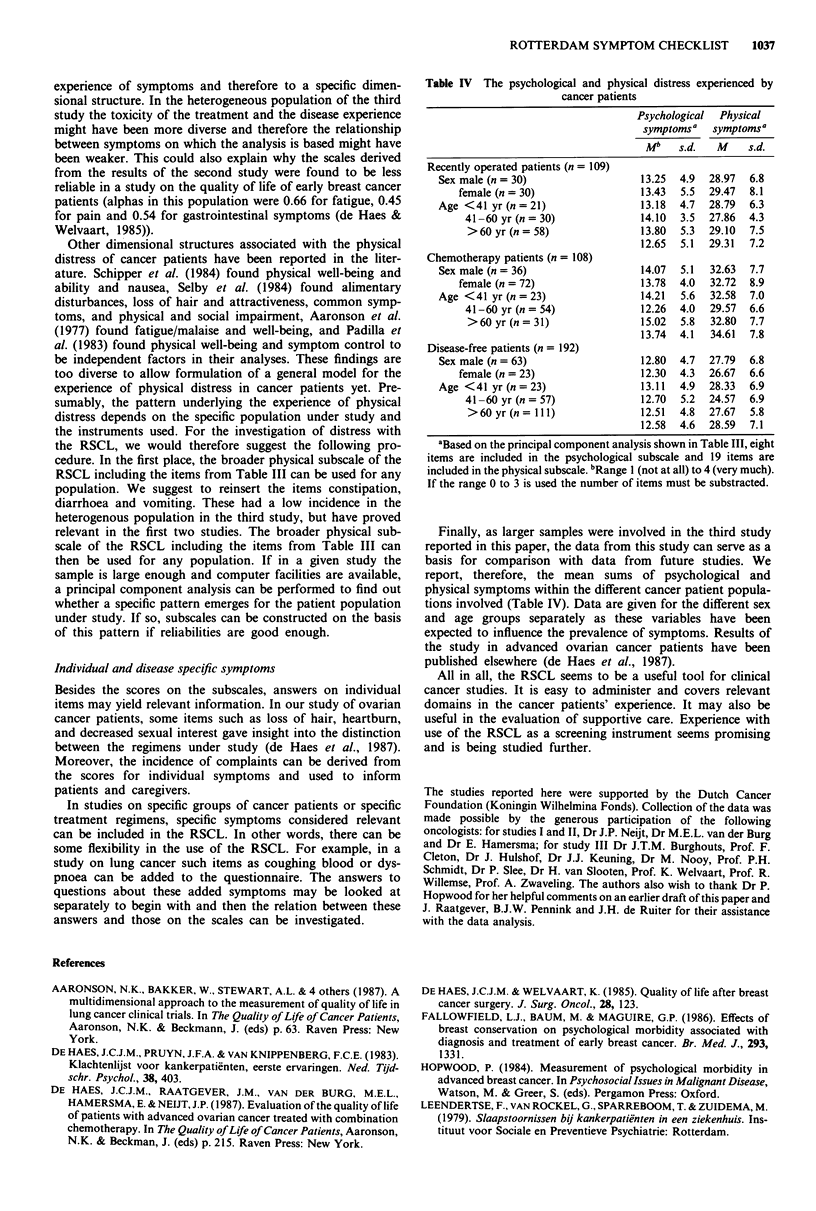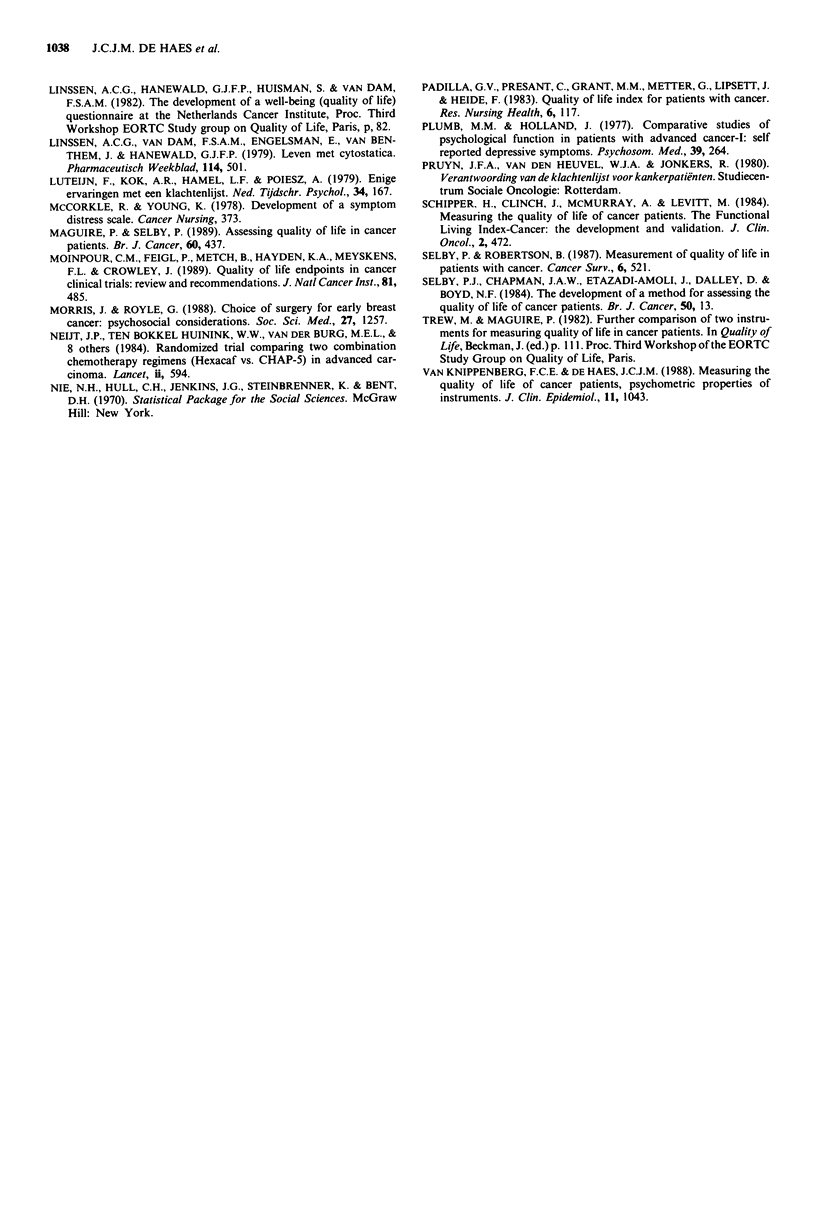# Measuring psychological and physical distress in cancer patients: structure and application of the Rotterdam Symptom Checklist.

**DOI:** 10.1038/bjc.1990.434

**Published:** 1990-12

**Authors:** J. C. de Haes, F. C. van Knippenberg, J. P. Neijt

**Affiliations:** Working Group on Medical Decision Making/Department of Clinical Oncology, University of Leiden, The Netherlands.

## Abstract

Use of the Rotterdam Symptom Checklist (RSCL) to measure psychological and physical distress as experienced by cancer patients, is discussed in this paper. The stability of the structure of the RSCL was assessed in principal component analyses in three studies: one concerning cancer patients during either chemotherapy or follow-up (n = 86), one done in patients undergoing chemotherapy for advanced ovarian cancer (n = 56), and the third dealing with cancer patients under treatment, disease-free 'patients', and 'normal' controls (n = 611). The psychological dimension proved to be stable across populations. A scale based on this factor was highly reliable (Cronbach's alpha 0.88-0.94). The physical distress is reflected by several dimensions in a homogeneous population (pain, fatigue, gastrointestinal complaints) and undimensionally in a heterogeneous population. Reliability of the physical distress scales is good (0.71-0.88). The current components of the RSCL and the use of individual and disease specific symptoms are discussed.


 © Macmillan Press Ltd., 1990

### Measuring psychological and physical distress in cancer patients: structure and application of the Rotterdam Symptom Checklist

J.C.J.M. de Haes[1], F.C.E. van Knippenberg[2] & J.P. Neijt[3]

[1]*Working Group on Medical Decision Making/Department of Clinical Oncology, University of Leiden and Department of Medical Psychology, University of Amsterdam;* [2]*Department of Medical Psychology and Psychotherapy, Erasmus University, Rotterdam; and* [3]*Department of Oncology, Utrecht University Hospital, Utrecht, The Netherlands*

**Summary** Use of the Rotterdam Symptom Checklist (RSCL) to measure psychological and physical distress as experienced by cancer patients, is discussed in this paper. The stability of the structure of the RSCL was assessed in principal component analyses in three studies: one concerning cancer patients during either chemotherapy or follow-up ($n = 86$), one done in patients undergoing chemotherapy for advanced ovarian cancer ($n = 56$), and the third dealing with cancer patients under treatment, disease-free 'patients', and 'normal' controls ($n = 611$). The psychological dimension proved to be stable across populations. A scale based on this factor was highly reliable (Cronbach's alpha 0.88–0.94). The physical distress is reflected by several dimensions in a homogeneous population (pain, fatigue, gastrointestinal complaints) and unidimensionally in a heterogeneous population. Reliability of the physical distress scales is good (0.71–0.88). The current components of the RSCL and the use of individual and disease specific symptoms are discussed.

During the past decade several instruments have been devised for measurement of the quality of life of cancer patients (for reviews see Selby & Robertson, 1987; van Knippenberg & de Haes, 1988; Maguire & Selby, 1989; Moinpour *et al.*, 1989). One of these instruments is called the Rotterdam Symptom Checklist (RSCL). The RSCL was developed, primarily, as a tool to measure the symptoms reported by cancer patients participating in clinical research. It is also applicable to monitor the levels of the patient's anxiety and depression and reflects the presence of psychological illness (Trew & Maguire, 1982).

The RSCL was constructed on the basis of analyses of the data from three studies done with different checklists (Pruyn *et al.*, 1980): (1) the Hopkins Symptom Checklist, which was used in a population of 352 psychiatric patients, 147 patients with rheumatoid arthritis, and 308 'normal' controls (Luteijn *et al.*, 1979); (2) a symptom checklist used in a study on the symptoms of 150 breast cancer patients (Linssen *et al.*, 1979); and (3) a Dutch version of the Symptom Distress Scale developed by McCorkle and Young (1978) applied to a group of 49 hospitalised cancer patients (Leendertse *et al.*, 1979). The initial selection of items from these checklists was based on factor loadings, relevance according to a group of experts in oncology, and the distribution of answers. Items with excessively skewed distributions were excluded. This yielded a 34-item list comprising physical and psychological symptoms (given in Table I). Patients were asked to indicate the degree to which they had been bothered by the indicated symptoms during the past three days, on a four-point, Likert-type rating scale (categories: not at all, a little, quite a bit, very much). The format is given in Figure 1. Eight items referring to the activities of daily living were added to cover the patient's functional status. Completion of the RSCL takes about 8 minutes.

In this questionnaire you are asked about your symptoms. Would you please, for any of the symptoms mentioned, indicate to what extent you have been bothered by it, by circling the answer most applicable to you. The questions are related to the past 3 days (the past week).

Have you, during the last 3 days (week), been bothered by

| | | | | |
|---|---|---|---|---|
| lack of appetite: | not at all | a little | quite a bit | very much |
| irritability: | not at all | a little | quite a bit | very much |
| tiredness: | not at all | a little | quite a bit | very much |
| worrying: | etc. | | | |

A current British version used in studies performed by the CRC Psychological Medicine Group in Manchester (Director: G.P. Maguire) has a slightly different format:

| Lack of appetite | | Irritability | | etc. |
|---|---|---|---|---|
| not at all | ☐ | not at all | ☐ | |
| a little | ☐ | a little | ☐ | |
| somewhat | ☐ | somewhat | ☐ | |
| very much | ☐ | very much | ☐ | |

**Figure 1** The format of the symptom checklist in the RSCL.

**Table I** Factor structure[a] and factor loadings (>0.40) after Varimax rotation of 34 symptoms in a population of female cancer patients attending an outpatient clinic ($n = 86$)

| | *Factor 1* | *Factor 2* | *Factor 3* | *Factor 4* |
|---|---|---|---|---|
| Lack of appetite | – | – | 0.54 | 0.42 |
| Irritability | – | – | 0.55 | – |
| Tiredness | – | – | – | 0.56 |
| Worrying | 0.81 | – | – | – |
| Sore muscles | – | 0.70 | – | – |
| Depressed mood | 0.77 | – | – | – |
| Lack of energy | – | – | – | 0.71 |
| Low back pain | – | 0.66 | – | – |
| Nervousness | 0.64 | – | – | – |
| Nausea | – | – | 0.64 | – |
| Desperate feelings about the future | 0.77 | – | – | – |
| Difficulties sleeping | – | – | – | – |
| Headaches | – | 0.40 | – | – |
| Vomiting | – | – | 0.89 | – |
| Dizziness | – | 0.45 | – | – |
| Decreased sexual interest | – | – | – | – |
| Itching | – | – | – | – |
| Feeling lonely | 0.65 | – | – | – |
| Tension | 0.78 | – | – | – |
| Crying spells | 0.56 | – | – | – |
| Abdominal aches | – | – | – | – |
| Anxiety | 0.85 | – | – | – |
| Constipation | – | 0.47 | – | – |
| Diarrhoea | – | – | – | – |
| Heartburn/belching | – | – | 0.42 | – |
| Shivering | – | 0.58 | – | – |
| Tingling hands or feet | – | – | – | – |
| Difficulty concentrating | 0.51 | – | – | – |
| Sore mouth/pain when swallowing | – | – | – | – |
| Loss of hair | – | – | – | – |
| Burning (or sore) eyes | – | – | – | – |
| Deafness | – | – | – | – |
| Shortness of breath | – | 0.62 | – | – |
| Dry mouth | – | – | – | 0.54 |

[a]Explaining 39.4% of the variance before rotation.

Correspondence: J.C.J.M. de Haes, Department of Medical Psychology, University Hospital (AMC), Meibergdreef 9, 1105 AZ Amsterdam, The Netherlands.
Received 10 April 1990; and in revised form 30 July 1990.

The RSCL was originally validated in a Dutch study (de Haes *et al.*, 1983) and since then has been used in a number of Dutch and British investigations (Hopwood, 1984; de Haes & Welvaart, 1985; Fallowfield *et al.*, 1986; Morris & Royle, 1988), which have provided experience with its application. This paper describes the principal component analyses of the symptom checklist done in three different studies, which established the stability of the structure of the RSCL, and it also deals with the reliability of subscales. Finally, the use of individual and disease-specific items will be discussed.

### Patients and methods

The structure of the RSCL and its stability were investigated in three studies. For validation of the instrument, it was first given to a sample of 95 female cancer patients visiting an outpatient clinic for either chemotherapy or follow-up ($n = 86$, 10% refused participation). The patients were asked to fill in the questionnaire when waiting in the hospital and give it to their oncologist when visiting him or her.

The second of these studies was done in 56 patients participating in a randomised trial comparing two chemotherapy regimens, Hexacaf and CHAP-5, for the treatment of advanced ovarian cancer (Neijt *et al.*, 1984). These patients completed the questionnaire in the clinic several times in the course of the treatment (mean number completed: 5.3 and 7.5, respectively).

In the third study the quality of life of cancer patients was compared with that of a group of 'normal' controls. A pilot study ($n = 20$) was done to establish the relevance of the questionnaire. Next, two groups – a heterogenous group of cancer patients who had either been operated on in the past 3 months or were receiving chemotherapy, and a group of cancer patients who had been without symptoms of the disease for 3 years or more – were compared with a random sample of the Dutch population. Patients and controls were sent a letter inviting them to participate, a copy of the questionnaire, and a return envelope. The questionnaire was completed and returned by 78% of the patients currently under treatment ($n = 216$), 87% of the disease-free 'patients' ($n = 192$) and 72% of the normal controls ($n = 201$).

### *Analysis*

To investigate the pattern underlying the experience of symptoms, we subjected the collected symptom scores to a principal component analysis in all three studies. This analysis permits the detection of independent dimensions (factors) in a set of items on the basis of their inter-relations. The factor solution chosen for the analyses presented here is based on the eigenvalues ($>1.0$) and the interpretability of the factors. For all analyses use was made of the Statistical Package for the Social Sciences (Nie *et al.*, 1970). In the second study, a mean score per item per person was computed across questionnaire administration, because the number of completed questionnaires was not the same for all patients. These mean scores were included in the analysis. For the third study the principal component analysis was carried out for the three groups of subjects together, to permit comparison of these groups on the basis of the scales to be constructed subsequently.

On the basis of the factors identified, subscales in the RSCL were constructed. The scores (range 1–4) on the different items found to load on the factors were added. In due course, the reliability of these scales was assessed with the use of Cronbach's alpha.

### Results

In the first study a four factor structure was found. The factor loadings of the symptoms on these factors are given in Table I. As this table shows, the first factor (explaining 22.7% of the variance) refers to a psychological dimension. All items with high loadings describe an element of the experience of psychological distress. The highest loading on the second factor (explaining 7.8% of the variance) have sore muscles and pain in the back. These symptoms, as well as headaches, refer to the experience of pain. A number of symptoms, such as shortness of breath, constipation, shivering and dizziness are correlated with this factor, but their content is less directly related to the experience of pain. The third factor (explaining 5.0% of the variance) refers to the experience of gastrointestinal complaints: vomiting, nausea and lack of appetite load highly on this factor. The loading of irritability on this factor is difficult to explain. On the fourth factor (explaining 3.9% of the variance) fatigue and lack of energy are important items. Lack of appetite is weakly related to this factor. The pattern emerging from this analysis is more or less unambiguously concerned with relevant elements in the experience of the disease and treatment. The content of the first factor, psychological distress, is the clearest, that of the others factors, i.e. pain, gastrointestinal complaints, and fatigue, is less distinct.

The results of the principal component analysis performed for the second study are given in Table II. Because the number of patients was rather low ($n = 56$), symptoms with a low mean score for the whole group (mean $<1.50$ (range 1–4)) and weakly related to other symptoms (Pearson correlation $<0.40$) were excluded from the analysis. Twenty-two items were included. Table II shows the factor loadings of 0.40 or higher.

Four factors again had eigenvalues higher than 1 and gave an interpretable solution. These factors explained 42.2, 9.5, 8.4 and 6.4% of the variance. As before, the first factor is composed of the psychological items included in the RSCL.

**Table II** Factor structure[a] and factor loadings ($>0.40$) after Varimax rotation of 34 symptoms in a population of advanced ovarian cancer patients undergoing chemotherapy ($n = 56$)

| | *Factor 1* | *Factor 2* | *Factor 3* | *Factor 4* |
|---|---|---|---|---|
| Lack of appetite | – | – | 0.73 | – |
| Irritability | 0.53 | – | – | – |
| Tiredness | – | – | – | 0.60 |
| Worrying | 0.84 | – | – | – |
| Sore muscles | – | 0.66 | – | – |
| Depressed mood | 0.79 | – | – | – |
| Lack of energy | – | – | – | 0.76 |
| Low back pain | – | 0.65 | – | – |
| Nervousness | 0.62 | – | – | – |
| Nausea | – | – | 0.81 | – |
| Desperate feelings about the future | 0.84 | – | – | – |
| Difficulties sleeping | 0.63 | – | – | – |
| Headaches | – | 0.66 | – | – |
| Vomiting | – | – | 0.95 | – |
| Dizziness | – | – | – | – |
| Decreased sexual interest | – | – | – | – |
| Itching | – | – | – | – |
| Feeling lonely | 0.68 | – | – | – |
| Tension | 0.77 | – | – | – |
| Crying spells | 0.43 | – | – | – |
| Abdominal aches | – | 0.64 | – | – |
| Anxiety | 0.88 | – | – | – |
| Constipation | – | 0.60 | – | – |
| Diarrhoea | – | – | – | – |
| Heartburn/belching | – | – | – | – |
| Shivering | – | – | – | 0.46 |
| Tingling hands or feet | – | – | – | – |
| Difficulty concentrating | – | – | – | – |
| Sore mouth/pain when swallowing | – | – | – | – |
| Loss of hair | – | – | – | – |
| Burning (or sore) eyes | – | – | – | – |
| Deafness | – | – | – | – |
| Shortness of breath | – | – | – | – |
| Dry mouth | – | – | – | – |

[a]Explaining 66.5% of the variance before rotation (the factor loadings and the variance explained in this analysis may be greater because of the smaller sample size and the use of aggregated data). [b]Loading of this item did not reach 0.40 for either the psychological or the physical dimension.

The second factor almost exclusively concerns items referring to the experience of pain in different parts of the body. The third factor refers to gastrointestinal symptoms and the fourth factor to the experience of fatigue and malaise. In this second study these factors are less ambiguous than in the first study: all symptoms are, at face value, related to the concent of the different factors.

Based on these results, subscales were defined for use in the comparison of the chemotherapy regimens; these pertained to psychological distress, pain, gastrointestinal symptoms and fatigue and proved to have good reliabilities (Cronbach's alphas 0.94, 0.81, 0.88, and 0.72 respectively).

The findings in these first two studies led to a proposal for a revised version of the RSCL (de Haes *et al.*, 1983). Firstly, the time period for symptom reporting was changed. After the first two studies were conducted, some empirical evidence was reported in the literature indicating that a period of 1 week was short enough to be remembered easily and not influenced in a substancial way by patients' 'complaining tendencies' (Linssen *et al.*, 1982). Secondly, some items were changed. Four symptoms were excluded because of low incidence or low factor loadings, i.e. itching, crying spells, concentration problems and deafness. The item difficulty sleeping was replaced by two items: awaking with a start and difficulty falling asleep. The revised list had 31 items. However, the pilot investigation for the third study led to some further changes once more. Concentration problems were mentioned spontaneously by chemotherapy patients and were, therefore, reinserted. The answers to the two new sleeplessness items turned out to have a very skewed distribution and these questions were replaced by the original one. The items feeling lonely, constipation, diarrhoea and vomiting had very low mean scores (mean $<1.2$) and were omitted.

The results of the third principal component analysis are presented in Table III. These factors explained 35.5% of the variance. The first factor (explaining 27.4% of the variance) refers evidently to the experience of psychological distress. In contrast to the earlier analyses, lack of energy is moderately and headaches and decreased sexual interest are weakly related to this factor. All other items that at face value would be considered psychological were related to this factor, often strongly. The second factor (explaining 8.1% of the variance) concerns almost all of the physical symptoms in the checklist. A number of symptoms are weakly related to this factor (sore muscles, low back pain, abdominal aches).

Based on the results of this analysis, a psychological distress scale and a physical distress scale were constructed by adding the scores of respondents on the relevant symptoms. All items included in the analysis were incorporated into these scales. The reliability of both the psychological and the physical distress scale was high (Cronbach's alpha 0.88 and 0.82, respectively).

**Table III** Factor structure[a] and factor loadings ($>0.40$) after Varimax rotation of 27 symptoms in cancer patients and normal controls ($n = 611$)

| | *Factor 1* | *Factor 2* |
|---|---|---|
| Lack of appetite | – | 0.51 |
| Irritability (ps)[b] | 0.66 | – |
| Tiredness | 0.40 | 0.57 |
| Worrying (ps) | 0.83 | – |
| Sore muscles | – | – |
| Depressed mood (ps) | 0.81 | – |
| Lack of energy | 0.58 | 0.44 |
| Low back pain | – | – |
| Nervousness (ps) | 0.72 | – |
| Nausea | – | 0.61 |
| Desperate feelings about the future (ps) | 0.67 | – |
| Difficulties sleeping | 0.54 | – |
| Headaches | (0.32) | – |
| Vomiting | – | –[c] |
| Dizziness | – | 0.43 |
| Decreased sexual interest | (0.31) | – |
| Tension (ps) | 0.77 | – |
| Abdominal ache | – | – |
| Anxiety (ps) | 0.75 | – |
| Constipation | – | –[c] |
| Diarrhoea | – | –[c] |
| Heartburn/belching | – | 0.52 |
| Shivering | – | 0.56 |
| Tingling hands or feet | – | 0.41 |
| Difficulty concentrating (ps) | 0.57 | – |
| Sore mouth/pain when swallowing | – | 0.54 |
| Loss of hair | – | 0.52 |
| Burning (or sore) eyes | – | 0.42 |
| Shortness of breath | – | 0.44 |
| Dry mouth | – | 0.66 |

[a]Explaining 35.5% of the variance before rotation. [b]The items followed by (ps) are included in the psychological distress scale, the other items in the physical distress scale. [c]Excluded because of the skewed distribution of answers.

### Discussion

The analysis of the structure of the RSCL showed that the psychological and the physical dimensions are both essential in the symptom experience of cancer patients and can be distinguished empirically.

### *Psychological distress*

A psychological dimension covering the psychological distress experienced by cancer patients is clearly discernable in all three of the analyses reported here. It seems to be a stable element in the structure of the RSCL. The reliability of the scale constructed on the basis of the results was consistently high.

The items included in the psychological factor were not always the same. In the first study, irritability belonged to the gastrointestinal factor. This is difficult to explain. Even more interesting is the finding that lack of energy and headaches loaded on the physical subscales in the homogeneous cancer patient population, and more clearly on the psychological dimension in the heterogeneous population. Also, the item difficulty sleeping loaded less consistently on the psychological subscale. These symptoms may be considered more or less psychosomatic. A similar mechanism has been described by Plumb and Holland (1977). They suggested that the physical symptoms that usually accompany psychological morbidity have a different meaning for cancer patients. These symptoms are probably related to the disease or an effect of the cancer treatment. Therefore, they should not be included in instruments designed to measure anxiety and depression. This assumption is supported by the results of the studies reported here. On these grounds it seems preferable to include only purely psychological items in the psychological distress subscale of the RSCL. If this is done, the psychological subscale of the RSCL would, as indicated in Table III, contain eight items: irritability, worrying, depressed mood, nervousness, desperate feelings about the future, tension, anxiety and problems concentrating. Reliability analysis points out that the alpha of this scale in the third study would be 0.89.

It is interesting to note, that the psychological distress experienced by patients is to a high degree independent of their physical distress. Psychological symptoms do not automatically accompany physical distress. Neither do they occur more intensely when less physical distress is experienced less during the illness process.

### *Physical distress*

The pattern underlying the experience of physical distress is less stable. In the first and the second studies we found three factors tapping different symptom areas: pain, fatigue, and gastrointestinal complaints. This distinction did not become evident in the third study. This difference might be explained by the homogeneity of the populations studied. In the first two studies, most participating patients received chemotherapy. This treatment may lead to a distinct pattern in the experience of symptoms and therefore to a specific dimensional structure. In the heterogeneous population of the third study the toxicity of the treatment and the disease experience might have been more diverse and therefore the relationship between symptoms on which the analysis is based might have been weaker. This could also explain why the scales derived from the results of the second study were found to be less reliable in a study on the quality of life of early breast cancer patients (alphas in this population were 0.66 for fatigue, 0.45 for pain and 0.54 for gastrointestinal symptoms (de Haes & Welvaart, 1985)).

Other dimensional structures associated with the physical distress of cancer patients have been reported in the literature. Schipper *et al.* (1984) found physical well-being and ability and nausea, Selby *et al.* (1984) found alimentary disturbances, loss of hair and attractiveness, common symptoms, and physical and social impairment, Aaronson *et al.* (1977) found fatigue/malaise and well-being, and Padilla *et al.* (1983) found physical well-being and symptom control to be independent factors in their analyses. These findings are too diverse to allow formulation of a general model for the experience of physical distress in cancer patients yet. Presumably, the pattern underlying the experience of physical distress depends on the specific population under study and the instruments used. For the investigation of distress with the RSCL, we would therefore suggest the following procedure. In the first place, the broader physical subscale of the RSCL including the items from Table III can be used for any population. We suggest to reinsert the items constipation, diarrhoea and vomiting. These had a low incidence in the heterogenous population in the third study, but have proved relevant in the first two studies. The broader physical subscale of the RSCL including the items from Table III can then be used for any population. If in a given study the sample is large enough and computer facilities are available, a principal component analysis can be performed to find out whether a specific pattern emerges for the patient population under study. If so, subscales can be constructed on the basis of this pattern if reliabilities are good enough.

*Individual and disease specific symptoms*

Besides the scores on the subscales, answers on individual items may yield relevant information. In our study of ovarian cancer patients, some items such as loss of hair, heartburn, and decreased sexual interest gave insight into the distinction between the regimens under study (de Haes *et al.*, 1987). Moreover, the incidence of complaints can be derived from the scores for individual symptoms and used to inform patients and caregivers.

In studies on specific groups of cancer patients or specific treatment regimens, specific symptoms considered relevant can be included in the RSCL. In other words, there can be some flexibility in the use of the RSCL. For example, in a study on lung cancer such items as coughing blood or dyspnoea can be added to the questionnaire. The answers to questions about these added symptoms may be looked at separately to begin with and then the relation between these answers and those on the scales can be investigated.

**Table IV** The psychological and physical distress experienced by cancer patients

| | *Psychological symptoms*[a] | | *Physical symptoms*[a] | |
|---|---|---|---|---|
| | *M*[b] | *s.d.* | *M* | *s.d.* |
| Recently operated patients ($n = 109$) | | | | |
| Sex male ($n = 30$) | 13.25 | 4.9 | 28.97 | 6.8 |
| female ($n = 30$) | 13.43 | 5.5 | 29.47 | 8.1 |
| Age <41 yr ($n = 21$) | 13.18 | 4.7 | 28.79 | 6.3 |
| 41–60 yr ($n = 30$) | 14.10 | 3.5 | 27.86 | 4.3 |
| >60 yr ($n = 58$) | 13.80 | 5.3 | 29.10 | 7.5 |
| | 12.65 | 5.1 | 29.31 | 7.2 |
| Chemotherapy patients ($n = 108$) | | | | |
| Sex male ($n = 36$) | 14.07 | 5.1 | 32.63 | 7.7 |
| female ($n = 72$) | 13.78 | 4.0 | 32.72 | 8.9 |
| Age <41 yr ($n = 23$) | 14.21 | 5.6 | 32.58 | 7.0 |
| 41–60 yr ($n = 54$) | 12.26 | 4.0 | 29.57 | 6.6 |
| >60 yr ($n = 31$) | 15.02 | 5.8 | 32.80 | 7.7 |
| | 13.74 | 4.1 | 34.61 | 7.8 |
| Disease-free patients ($n = 192$) | | | | |
| Sex male ($n = 63$) | 12.80 | 4.7 | 27.79 | 6.8 |
| female ($n = 23$) | 12.30 | 4.3 | 26.67 | 6.6 |
| Age <41 yr ($n = 23$) | 13.11 | 4.9 | 28.33 | 6.9 |
| 41–60 yr ($n = 57$) | 12.70 | 5.2 | 24.57 | 6.9 |
| >60 yr ($n = 111$) | 12.51 | 4.8 | 27.67 | 5.8 |
| | 12.58 | 4.6 | 28.59 | 7.1 |

[a]Based on the principal component analysis shown in Table III, eight items are included in the psychological subscale and 19 items are included in the physical subscale. [b]Range 1 (not at all) to 4 (very much). If the range 0 to 3 is used the number of items must be substracted.

Finally, as larger samples were involved in the third study reported in this paper, the data from this study can serve as a basis for comparison with data from future studies. We report, therefore, the mean sums of psychological and physical symptoms within the different cancer patient populations involved (Table IV). Data are given for the different sex and age groups separately as these variables have been expected to influence the prevalence of symptoms. Results of the study in advanced ovarian cancer patients have been published elsewhere (de Haes *et al.*, 1987).

All in all, the RSCL seems to be a useful tool for clinical cancer studies. It is easy to administer and covers relevant domains in the cancer patients' experience. It may also be useful in the evaluation of supportive care. Experience with use of the RSCL as a screening instrument seems promising and is being studied further.

The studies reported here were supported by the Dutch Cancer Foundation (Koningin Wilhelmina Fonds). Collection of the data was made possible by the generous participation of the following oncologists: for studies I and II, Dr J.P. Neijt, Dr M.E.L. van der Burg and Dr E. Hamersma; for study III Dr J.T.M. Burghouts, Prof. F. Cleton, Dr J. Hulshof, Dr J.J. Keuning, Dr M. Nooy, Prof. P.H. Schmidt, Dr P. Slee, Dr H. van Slooten, Prof. K. Welvaart, Prof. R. Willemse, Prof. A. Zwaveling. The authors also wish to thank Dr P. Hopwood for her helpful comments on an earlier draft of this paper and J. Raatgever, B.J.W. Pennink and J.H. de Ruiter for their assistance with the data analysis.

**References**

AARONSON, N.K., BAKKER, W., STEWART, A.L. & 4 others (1987). A multidimensional approach to the measurement of quality of life in lung cancer clinical trials. In *The Quality of Life of Cancer Patients*, Aaronson, N.K. & Beckmann, J. (eds) p. 63. Raven Press: New York.

DE HAES, J.C.J.M., PRUYN, J.F.A. & VAN KNIPPENBERG, F.C.E. (1983). Klachtenlijst voor kankerpatiënten, eerste ervaringen. *Ned. Tijdschr. Psychol.*, **38**, 403.

DE HAES, J.C.J.M., RAATGEVER, J.M., VAN DER BURG, M.E.L., HAMERSMA, E. & NEIJT, J.P. (1987). Evaluation of the quality of life of patients with advanced ovarian cancer treated with combination chemotherapy. In *The Quality of Life of Cancer Patients*, Aaronson, N.K. & Beckman, J. (eds) p. 215. Raven Press: New York.

DE HAES, J.C.J.M. & WELVAART, K. (1985). Quality of life after breast cancer surgery. *J. Surg. Oncol.*, **28**, 123.

FALLOWFIELD, L.J., BAUM, M. & MAGUIRE, G.P. (1986). Effects of breast conservation on psychological morbidity associated with diagnosis and treatment of early breast cancer. *Br. Med. J.*, **293**, 1331.

HOPWOOD, P. (1984). Measurement of psychological morbidity in advanced breast cancer. In *Psychosocial Issues in Malignant Disease*, Watson, M. & Greer, S. (eds). Pergamon Press: Oxford.

LEENDERTSE, F., VAN ROCKEL, G., SPARREBOOM, T. & ZUIDEMA, M. (1979). *Slaapstoornissen bij kankerpatiënten in een ziekenhuis*. Instituut voor Sociale en Preventieve Psychiatrie: Rotterdam.

LINSSEN, A.C.G., HANEWALD, G.J.F.P., HUISMAN, S. & VAN DAM, F.S.A.M. (1982). The development of a well-being (quality of life) questionnaire at the Netherlands Cancer Institute, Proc. Third Workshop EORTC Study group on Quality of Life, Paris, p, 82.

LINSSEN, A.C.G., VAN DAM, F.S.A.M., ENGELSMAN, E., VAN BENTHEM, J. & HANEWALD, G.J.F.P. (1979). Leven met cytostatica. *Pharmaceutisch Weekblad*, **114**, 501.

LUTEIJN, F., KOK, A.R., HAMEL, L.F. & POIESZ, A. (1979). Enige ervaringen met een klachtenlijst. *Ned. Tijdschr. Psychol.*, **34**, 167.

McCORKLE, R. & YOUNG, K. (1978). Development of a symptom distress scale. *Cancer Nursing*, 373.

MAGUIRE, P. & SELBY, P. (1989). Assessing quality of life in cancer patients. *Br. J. Cancer*, **60**, 437.

MOINPOUR, C.M., FEIGL, P., METCH, B., HAYDEN, K.A., MEYSKENS, F.L. & CROWLEY, J. (1989). Quality of life endpoints in cancer clinical trials: review and recommendations. *J. Natl Cancer Inst.*, **81**, 485.

MORRIS, J. & ROYLE, G. (1988). Choice of surgery for early breast cancer: psychosocial considerations. *Soc. Sci. Med.*, **27**, 1257.

NEIJT, J.P., TEN BOKKEL HUININK, W.W., VAN DER BURG, M.E.L., & 8 others (1984). Randomized trial comparing two combination chemotherapy regimens (Hexacaf vs. CHAP-5) in advanced carcinoma. *Lancet*, **ii**, 594.

NIE, N.H., HULL, C.H., JENKINS, J.G., STEINBRENNER, K. & BENT, D.H. (1970). *Statistical Package for the Social Sciences*. McGraw Hill: New York.

PADILLA, G.V., PRESANT, C., GRANT, M.M., METTER, G., LIPSETT, J. & HEIDE, F. (1983). Quality of life index for patients with cancer. *Res. Nursing Health*, **6**, 117.

PLUMB, M.M. & HOLLAND, J. (1977). Comparative studies of psychological function in patients with advanced cancer-I: self reported depressive symptoms. *Psychosom. Med.*, **39**, 264.

PRUYN, J.F.A., VAN DEN HEUVEL, W.J.A. & JONKERS, R. (1980). *Verantwoording van de klachtenlijst voor kankerpatiënten*. Studiecentrum Sociale Oncologie: Rotterdam.

SCHIPPER. H., CLINCH, J., MCMURRAY, A. & LEVITT, M. (1984). Measuring the quality of life of cancer patients. The Functional Living Index-Cancer: the development and validation. *J. Clin. Oncol.*, **2**, 472.

SELBY, P. & ROBERTSON, B. (1987). Measurement of quality of life in patients with cancer. *Cancer Surv.*, **6**, 521.

SELBY, P.J., CHAPMAN, J.A.W., ETAZADI-AMOLI, J., DALLEY, D. & BOYD, N.F. (1984). The development of a method for assessing the quality of life of cancer patients. *Br. J. Cancer*, **50**, 13.

TREW, M. & MAGUIRE, P. (1982). Further comparison of two instruments for measuring quality of life in cancer patients. In *Quality of Life*, Beckman, J. (ed.) p. 111. Proc. Third Workshop of the EORTC Study Group on Quality of Life, Paris.

VAN KNIPPENBERG, F.C.E. & DE HAES, J.C.J.M. (1988). Measuring the quality of life of cancer patients, psychometric properties of instruments. *J. Clin. Epidemiol.*, **11**, 1043.